# Voyages to map unexplored parts of the epitranscriptomic world

**DOI:** 10.1038/s12276-022-00825-w

**Published:** 2022-10-21

**Authors:** Ki-Jun Yoon

**Affiliations:** Department of Biological Sciences and KAIST Stem Cell Center, Korea Advanced Institute of Science and Technology (KAIST), Daejeon, 34141 Republic of Korea

**Keywords:** Biological techniques, Sequencing

Nascent transcripts are subject to extensive processing, such as 5ʹ capping, splicing, 3ʹ polyadenylation, RNA editing, and RNA modifications. The transcriptome plasticity conferred by these posttranscriptional regulations can alter gene expression, which is a major driver of proteomic diversity. Similar to epigenetic modifications on DNA and histone proteins, RNA is also subject to various chemical modifications, called epitranscriptomic modifications. Since the first chemical modification of RNA was identified approximately 60 years ago, more than 170 RNA modifications have been identified, including N^6^-methyladenosine (m^6^A), pseudouridine (Ψ), 2ʹ-O-methylation, (Nm), 5-methylcytidine (m^5^C), and 8-oxoguanine (o^8^G). Different RNA species, such as ribosomal RNAs (rRNAs), transfer RNAs (tRNAs), messenger RNAs (mRNAs), and noncoding RNAs (ncRNAs), are frequently posttranscriptionally modified. In particular, studies in the past decade have suggested that these modifications influence almost all aspects of RNA metabolism, including stability, splicing, localization, RNA–protein interaction, and translation^1^. Indeed, these RNA modifications exhibit essential functions in many biological contexts, including normal development, adult tissue homeostasis, and cancer^2,3^.

Despite early biochemical studies showing the prevalence of chemically modified RNA in eukaryotes, the functional contribution of these marks to biological processes has been ignored for a long time. The discovery of molecular machinery that writes, erases and recognizes RNA modifications has helped in the characterization of the functional roles played by these modifications in various contexts. In addition, advances in transcriptome-wide detection methods have expanded our knowledge related to the precise position and abundance of RNA modifications in the transcriptome, leading to a greater understanding of epitranscriptomic regulation. Xiaoyu Li and colleagues reviewed advanced technologies used to detect epitranscriptomic marks on RNA^4^. To understand the functions of epitranscriptomic marks, information on the precise location and abundance of various chemical modifications in the transcriptome is critical. Multifaceted approaches, including thin-layer chromatography (TLC), liquid chromatography–mass spectrometry (LC-MS), and next-generation sequencing-based methods, have been developed. The aforementioned review summarizes the current knowledge on the history of the development, advantages, and limitations of RNA modification detection tools and discusses future challenges to a comprehensive understanding of the epitranscriptome.

Among the various RNA modifications, m^6^A is the most frequent internal mRNA mark and has been extensively studied in diverse cellular contexts. The epigenome and transcriptome in pluripotent stem cells (PSCs) undergo drastic changes during differentiation, and these changes are essential to control gene expression during the early development phase. In addition to genetic and epigenetic regulation, epitranscriptomic regulation shapes the expression profile of critical factors in the maintenance and fate specification of PSCs. For example, m^6^A RNA modification is essential for the formation of heterochromatin that silences retrotransposons, and this silencing is necessary to maintain embryonic stem cell identity^5,6^. In addition, the gene expression of developmental regulators during cell differentiation is controlled by m^6^A modification in embryonic stem cells^7^. Yong Jun Kim and colleagues reviewed gene expression control mediated through m^6^A and other RNA modification marks, especially in human PSCs and during early embryonic development^8^.

m^6^A marks are deposited on other RNA species, including rRNAs. Caroline Vissers and a colleague summarized the current knowledge of m^6^A modification on 18 S rRNAs, which is mediated by the methyltransferase METTL5^9^. In particular, m^6^A methylation of rRNAs regulates ribosomal activity by shaping the structure of the ribosomal decoding center, thus influencing protein translation efficiency. Interestingly, m^6^A abundance on rRNAs shows variability among cell types and species, suggesting that the m^6^A rRNA mark may participate in the dynamic regulation of protein translation in a context-dependent manner. In addition, mutations in the human *METTL5* gene are associated with the risk for intellectual disability and developmental abnormalities, suggesting potential roles played by rRNA m^6^A modification in neurodevelopment.

Reactive oxygen species affect various cellular processes by oxidizing biomolecules, including guanine bases in DNA and RNA. The resultant 8-oxoguanine in DNA (8-oxo-dG) can pair with adenine, causing a mutation (G > T) that is prevalent in the cancer genome, and this mutation is actively repaired by DNA repair pathways. In addition to damaging DNA, 8-oxo-dG is an epigenetic mark that changes gene expression by altering promoter activity and the distribution of histone modifications. In addition, guanine in RNA is frequently oxidized to produce o^8^G. Inappropriate o^8^G•A base pairing impacts RNA structure and functions at the posttranscriptional level, such as increasing the number of translational errors and reducing protein synthesis. Many studies have suggested that o^8^G can also alter RNA–RNA interactions in a redox-dependent manner. For example, a position-specific o^8^G mark on microRNA (miRNA) may be an epitranscriptomic mechanism that coordinate pathophysiological redox-mediated gene expression in cardiac hypertrophy^10^. In this special issue, Sung Wook Chi and colleagues present an overview of the function of 8-oxoguanine in DNA and RNA, from oxidative damage to the regulatory mechanisms of redox-mediated gene expression in both an epigenomic and epitranscriptomic manner^11^.

Despite the extensive efforts and remarkable recent progress made in the field, we have only started to explore the vast unknown landscape of the posttranscriptional regulation of gene expression. To date, the scientific community has focused on the functional relevance of only a few types of epitranscriptomic modifications, including m^6^A, m^5^C, and Ψ. Moreover, several additional questions will be an exciting avenue of research in the future: How do these epitranscriptomic modifications distinctly regulate RNA metabolism in different cell types in the complex tissue milieu? Do different types of epitranscriptomic modifications coexist and engage in interplay to regulate the metabolism of the same RNA molecule? What are the dynamic properties of the modifications that govern regulatory pathways in response to environmental changes or pathological conditions? Do species differences in epitranscriptomic regulation offer insight into human evolution? Can we leverage the modulation of the epitranscriptome as a therapeutic intervention for various human disorders? Advanced technologies that enable the precise detection and manipulation of the epitranscriptome in diverse cellular systems will comprise the foundation needed to answer these questions.
